# Chondroprotective effects and mechanisms of resveratrol in advanced glycation end products-stimulated chondrocytes

**DOI:** 10.1186/ar3127

**Published:** 2010-09-08

**Authors:** Feng-Cheng Liu, Li-Feng Hung, Wan-Lin Wu, Deh-Ming Chang, Chuan-Yueh Huang, Jenn-Haung Lai, Ling-Jun Ho

**Affiliations:** 1 Graduate Institute of Medical Science, National Defense Medical Center, Neihu 114, Taipei, Taiwan, ROC; 2 Division of Rheumatology/Immunology & Allergy, Department of Medicine, Tri-Service General Hospital, National Defense Medical Center, Neihu 114, Taipei, Taiwan, ROC; 3 Institute of Cellular and System Medicine, National Health Research Institute, Zhunan, Miaoli County 350, Taiwan, ROC; 4 Graduate Institute of Basic Medical Science, PhD Program of Aging, China Medical University, Taichung 40402, Taiwan, ROC

## Abstract

**Introduction:**

Accumulation of advanced glycation end products (AGEs) in joints contributes to the pathogenesis of cartilage damage in osteoarthritis (OA). We aim to explore the potential chondroprotective effects of resveratrol on AGEs-stimulated porcine chondrocytes and cartilage explants.

**Methods:**

Chondrocytes were isolated from pig joints. Activation of the IκB kinase (IKK)-IκBα-nuclear factor-kappaB (NF-κB) and c-Jun N-terminal kinase (JNK)/extracellular signal-regulated kinase (ERK)-activator protein-1 (AP-1) pathways was assessed by electrophoretic mobility shift assay (EMSA), Western blot and transfection assay. The levels of inducible nitric oxide synthase (iNOS)-NO and cyclooxygenase-2 (COX-2)-prostaglandin E_2 _(PGE_2_) were measured by Western blot, Griess reaction or ELISA. The expression and enzyme activity of matrix metalloproteinase-13 (MMP-13) were determined by real time RT/PCR and gelatin zymography, respectively.

**Results:**

We show that AGEs-induced expression of iNOS and COX-2 and production of NO and PGE_2 _were suppressed by resveratrol. Such effects of resveratrol were likely mediated through inhibiting IKK-IκBα-NF-κB and JNK/ERK-AP-1 signaling pathways induced by AGEs. By targeting these critical signaling pathways, resveratrol decreased AGEs-stimulated expression and activity of MMP-13 and prevented AGEs-mediated destruction of collagen II. Histochemistry analysis further confirms that resveratrol could prevent AGEs-induced degradation of proteoglycan and aggrecan in cartilage explants.

**Conclusions:**

The present study reveals not only the effects and mechanisms regarding how resveratrol may protect cartilage from AGEs-mediated damage but also the potential therapeutic benefit of resveratrol in the treatment of OA.

## Introduction

Osteoarthritis (OA) is a highly age-related inflammatory process of joints. Aside from ageing, other factors such as obesity, trauma, genetic predisposition and endocrine factors contribute to the pathophysiological events observed in the process of cartilage and joint damage [1,2]. Although age has been recognized as the primary risk factor for the development of OA, the association between ageing and inflammation of the joints remains obscure. During the ageing process, there is a phenomenon characterized by post-translational modification of proteins by non-enzymatic glycation and this process results in accumulation of advanced glycation end products (AGEs) in many tissues, including joints [3,4]. The accumulated AGEs can reduce proteoglycan and collagen synthesis by chondrocytes and lead to stiffness and fragility of articular cartilage [5]. AGEs also induce the production of matrix metalloproteases (MMPs) that are responsible for causing cartilage degradation and joint damage [6]. In addition to its role in the ageing process, the receptor for AGE (RAGE) appears to play roles in various inflammatory disorders [7].

Resveratrol (trans-3, 4',5-trihydroxystilbene), a polyphenolic natural phytoalexin found in a variety of food products, with particularly high levels in grape skin and red wine, preserves many properties such as anti-inflammation, anti-cancer, anti-oxidant and cardio-protection [8-10]. Although limited clinical evidence is available in OA, the anti-inflammatory properties of resveratrol have been shown to suppress anterior cruciate ligament transaction-induced experimental OA in rabbits [11]. Recent studies demonstrate that resveratrol can inhibit interleukin-1 (IL-1)-induced expression of COX-2 and production of PGE_2 _through suppressing NF-κB activity in human chondrocytes [12]. Additionally, resveratrol is capable via, in part, its anti-oxidant property of preventing IL-1-induced and p53-induced chondrocyte apoptosis [13,14].

The accumulated studies already suggest the pathogenic roles of cytokines like IL-1 and tumor necrosis factor alpha (TNF-α) in OA development [15]. Along these cytokines-mediated inflammatory processes, the transcription factors like NF-κB and AP-1 have been considered to play critical roles. Regarding this, the results from other researchers and us indicate that comparable to both IL-1 and TNF-α, AGEs can activate NF-κB and AP-1 signaling pathways in chondrocytes [16,17]. Furthermore, our results also indicate the critical roles of COX-2-PGE_2 _and iNOS-NO pathways in AGEs-mediated damage of cartilage [17]. In this report, we are interested in determining whether resveratrol can be protective in AGEs-induced inflammation of chondrocytes and damage of cartilage. The results demonstrate that through inhibiting the activation of several critical molecules, resveratrol effectively attenuated AGEs-induced chondrocyte activation and cartilage damage.

## Materials and methods

### Reagents and antibodies

Polyclonal antisera against iNOS, COX-2 and IκBα were purchased from Santa Cruz Biotechnology (Santa Cruz, CA, USA). Polyclonal anti-collagen II antibodies were purchased from Chemicon International (Temecula, CA, USA). The antibodies recognizing ERK, JNK, unphosphorylated IKKα or phospho-IKKα/β were purchased from Cell Signaling (Danvers, MA, USA). Resveratrol (R5010) was purchased from Sigma-Aldrich Chemical Company (St. Louis, MO, USA) and was prepared as a 200 mM stock concentration with ethanol. The required concentrations of resveratrol for individual experiments were made by further dilution of the stock preparation with culture medium when needed. Unless otherwise specified, the rest of the reagents were purchased from Sigma-Aldrich Chemical Company.

### Preparation of AGEs

Methylglyoxal-modified albumin, as the source of AGEs examined in this study, was prepared as described [17]. In brief, by incubating 10 mg/ml of bovine serum albumin (BSA) (USB Corporation, Cleveland, OH, USA) in phosphate-buffered saline (PBS) containing 50 mM methylglyoxal, 0.1% sodium azide, and 1 mM phenylmethylsulphonyl fluoride (PMSF) at 37°C for seven days, the crude preparation of AGEs was obtained. After dialysis against PBS, the AGEs preparation was filtrated and stored at -80°C until use. The AGEs preparation using methylglyoxal-modified albumin contains N(epsilon)-carboxyethyl-lysine (CEL) and other AGEs [18]. To enhance the significance of this study, the AGEs preparation using glycoaldehyde-modified albumin that contains N(epsilon)-carboxymethyllysine (CML), pentosidine and other AGEs was also prepared according to the report [19,20].

### Isolation and culture of porcine chondrocytes

Porcine cartilage was obtained from the hind leg joints of pigs. The preparation of chondrocytes from cartilage was performed according to our previous report [17]. After enzymatic digestion of articular cartilage with 2 mg/ml protease in serum-free Dulbecco's modified Eagle's medium (DMEM) containing antibiotics and 10% fetal bovine serum (FBS), the specimens were then digested overnight with 2 mg/ml collagenase I and 0.9 mg/ml hyaluronidase in DMEM/antibiotics. The cells were collected, passed through a cell strainer (Beckton Dickinson, Mountain View, CA, USA) and cultured in DMEM containing 10% FBS and antibiotics for three to four days before use.

### Cytotoxicity analysis

Evaluation of potential cytotoxic effects of resveratral was performed by using 3-(4,-Dimethylthiazol-2-y)-2,5-diphenyl-tetrazolium bromide (MTT) colorimetric assays as described [21]. In brief, chondrocytes were incubated in the presence or absence of resveratrol for 48 h. Then, 25 μl of MTT (5 mg/ml in H_2_O) was added, and cells were incubated at 37°C for 2 h followed by the addition of 100 μl of lysis buffer containing 20% sodium dodecyl sulfate and 50% dimethylformamide. After incubation at 37°C for another 6 h, the content of dissolved reduced MTT crystals was measured with an ELISA reader (Dynatech, Chantilly, VA, USA).

### Transfection and luciferase assays

Porcine chondrocytes were seeded one day before the transfection experiment at a density of 3 × 10^5 ^cells/6-cm dish. TransIT^®^-LT1 transfection reagent (Pan-vera, Madison, WI, USA) was used to co-transfect 1.5 μg/ml of the reporter plasmid pNF-κB-luciferase or pAP-1-luciferase with the internal control plasmid pTK-Renilla-luciferase (Promega, Madison, WI, USA) at a ratio of 100:1 into cells in serum- and antibiotic-free DMEM. Four hours after transfection, the culture medium was replaced with fresh DMEM supplemented with 2% FBS. For luciferase assay, total cell lysates were prepared and the firefly luciferase activity was measured and normalized to Renilla luciferase activity according to the dual luciferase reporter system (Promega).

### Measurement of NO and PGE_2 _concentrations

The measurement of NO release was reflected by determination of its stable end product, nitrite, in supernatants [17]. The Griess reaction was performed with the concentrations of nitrite measured by a spectrophotometer. In brief, an aliquot (100 μl) of culture supernatant was incubated with 50 μl of 0.1% sulfanilamide in 5% phosphoric acid and 50 μl of 0.1% *N*-1-naphthyl-ethylenediamine dihydrochloride. After 10 minutes of incubation at room temperature, the absorbance was measured at 550 nm wavelength with a plate reader (Tecan, Grodig, Australia). PGE_2 _concentrations were determined by ELISA according to the manufacturer's protocol (Cayman, Ann Arbor, MI, USA).

### Western blotting

ECL Western blotting (Amersham-Pharmacia, Arlington Heights, IL, USA) was performed as described [22]. Briefly, equal amounts of whole cellular extracts were analyzed on 10% sodium dodecyl sulphate-polyacrylamide gel electrophoresis (SDS-PAGE) and transferred to the nitrocellulose filter. For immunoblotting, the nitrocellulose filter was incubated with Tris-buffered saline with 1% Triton X-100 containing 5% non-fat milk for 1 h and then blotted with antibodies against specific proteins for another 2 h at room temperature. After washing with milk buffer, the filter was incubated with rabbit anti-goat IgG or goat anti-rabbit IgG conjugated to horseradish peroxidase at a concentration of 1:5000 for 30 minutes. The filter was incubated with the substrate and then exposed to X-ray film (Kodak, Rochester, NY, USA).

### Nuclear extract preparation and electrophoretic mobility shift assay (EMSA)

Nuclear extract preparation and EMSA analysis were performed as detailed in our previous report [22]. The oligonucleotides containing NF-κB-binding site, SP-1 binding site or AP-1-binding site were purchased and used as DNA probes. The DNA probes were radio-labeled with (γ-^32^P)ATP using the T4 kinase (Promega). For the binding reaction, the radio-labeled pr)be was incubated with 4 μg of nuclear extracts. The binding buffer contained 10 mM Tris-HCl (pH 7.5), 50 mM NaCl, 0.5 mM ethylenediaminetetraacetic acid (EDTA), 1 mM dithiothreitol, 1 mM MgCl_2_, 4% glycerol and 2 μg poly(dI-dC). The reaction mixture was left at room temperature to proceed with binding reaction for 20 minutes. The final reaction mixture was analyzed in a 6% non-denaturing polyacrylamide gel with 0.5 × Tris/Borate/EDTA as an electrophoresis buffer.

### Gelatin zymography

Gelatin zymography was performed according to the description by other researchers [20,23]. The culture supernatant (16 μl) was mixed with 4 μl buffer containing 4% SDS, 0.15 M Tris (pH 6.8), and 20% glycerol that contains 0.05% bromophenol blue and analyzed on a 10% polyacrylamide gel with copolymerized 0.1% gelatin (Sigma-Aldrich). The positive control of MMP-13 was purchased (EMD Chemicals, Gibbstown, NJ, USA). After electrophoresis, gels were washed with 2.5% Triton X-100 for three times with 20 minutes each. After incubation with the gelatinase buffer (50 mM Tris-HCl (pH 7.6), 10 mM CaCl_2_, 50 mM NaCl, and 0.05% Brij-35) at 37°C for 24 h, the gel was stained with 0.1% Coomassie blue, and the clear bands that indicate gelatinolytic activity were visualized under the background of uniform light blue staining. The localization of MMP-13 was identified as judged by the molecular weight of the standards and the report from other researchers [24] under Casio Ex Z60 digital camera (Shibuy-ku, Tokyo, Japan).

### Immunoprecipitation kinase assay

This assay has been described in our previous work [22]. In brief, the whole cellular extract 50 to 100 μg was incubated overnight with 5 μl of specific antibodies in incubation buffer containing 25 mM HEPES (pH 7.7), 300 mM NaCl, 1.5 mM MgCl2, 0.2 mM EDTA, 0.1% Triton-X-100, 20 mM β-glycerophosphate, 0.1 mM Na3VO4, 2 μM leupeptin, and 400 μM PMSF. The mixture was then immunoprecipitated by the addition of protein A beads and rotated at 4°C for 2 h. After extensive wash, the beads were resuspended in 40 μl kinase buffer with addition of cold ATP (30 μM), 8 ng of substrate (GST-c-Jun), and 10 μCi of (γ-32P)ATP. The mixture was incubated at 30°C with occasional gentle mixing for 30 minutes. The reaction was then terminated by resuspending in 1% SDS solubilizing buffer and boiling for five minutes; then the samples were analyzed in SDS-PAGE.

### Real-time RT/PCR

Total RNA was isolated after lysing the cells by Trizol (Invitrogen, Carlsbad, CA, USA). After reverse transcription of RNA to cDNA, samples were subjected to PCR reactions. Real-time quantitation of MMP-13 and GAPDH messenger RNA (mRNA) was performed according to the manufacturer's instructions (CYBR Green Master Mix, Applied BioSystems, Foster City, CA, USA). In brief, 10 ng of cDNA was amplified in a total volume of 20 μl consisting of 1× Master Mix and the gene-specific primers, which were added at a final concentration of 100 n*M*. The primer sequences for both MMP-13 and GAPDH were described by other reports [25,26]: 5'-CCA AAG GCT ACA ACT TGT TTC TTG -3' and 5'-TGG GTC CTT GGA GTG GTC AA -3'(for MMP-13); 5'-TGT C AT CCA TGA CAA CTC GG -3' and 5'-GCC ACA GTT TCC CAG AGG -3' (for GAPDH). The reactions were performed for 50 cycles with 95°C for denaturation and 60°C for annealing and extension on the ABI 7300 real-time PCR system (Applied BioSystems). The data were collected and the fold changes in gene expression following stimulation in the presence or absence of resveratrol was calculated with the following formula: fold changes = $2^{-\Delta (\Delta {\text{C}}_{\text{t}})}$, where C_t _= C_t stimulated _- C_t GAPDH_, and (C_t_) = C_t stimulated _- C_t control_.

### Preparation of cartilage explants

The preparation of cartilage explants was performed according to our previous report with mild modification [17]. In brief, articular cartilage from the femur head of hind limb joint of pigs was excavated by a stainless-steel dermal-punch 3 mm in diameter (Aesculap, Tuttlingen, Germany) and weighed. After dissection, each cartilage explant was placed in a 24-well plate and cultured for 24 h in DMEM containing antibiotics and 10% FBS. After resting for 72 h in serum-free DMEM, the cartilage explants were used for further study.

### Analysis of cartilage degradation

Cartilage degradation was assessed by measuring the amount of proteoglycan released into the culture medium as described [17]. In brief, culture medium was added to 1,9-dimethylmethylene blue (DMB) solution (Sigma-Aldrich) in which the metachromatic dye can bind sulfated glycosaminoglycan (GAG), a major component of proteoglycan. The amount of the formation of GAG-DMB complex was measured in a 96-well plate using a plate reader (TECAN, Grödig, Austria) at a wavelength 595 nm. The loss of GAG was calculated and expressed as the total GAG (μg) released per mg (wet weight) of the cartilage weight.

### Safranin O staining and measurement of the aggrecan NITEGE neoepitope

Cartilage explants were mounted in embedding medium (Miles Laboratories, Naperville, IL, USA) and rapidly frozen at -80°C. Serial but incontinuous microscopic sections (7 μm) of cartilage explants were cut at -20°C on a Microm cryostat and mounted on Superfrost Plus glass slides (Menzel-Gläser, Braunschweig, Germany). Tissue sections were then stained with Safranin O/fast green countered with Weigert's iron hematoxylin to assess the changes of proteoglycan content [17]. In parallel, the expression of aggrecan NITEGE neoepitope recognized by NITEGE antibodies in tissue sections was determined as described by other researchers [27].

### Statistical analysis

When necessary, the results were expressed as mean ± SD. Unpaired Student's t-test was used to determine the difference, which was considered to be significant when *P *< 0.05.

## Results

### Resveratrol inhibited iNOS-NO and COX-2-PGE_2 _production in AGEs-stimulated porcine chondrocytes

**Figure 1:**
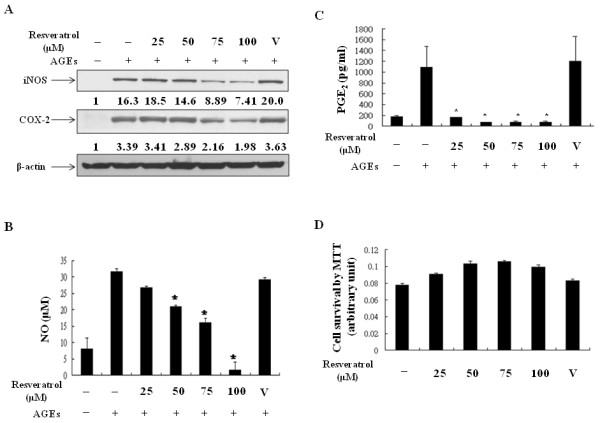
**Resveratrol inhibited AGEs-induced expression of iNOS and COX-2 and production of NO and PGE_2_**. Chondrocytes 1 × 10^6^/ml were pretreated with various doses of resveratrol or the solvent (V), ethanol, for 24 h and then stimulated with 100 μg/ml AGEs for another 24 h. The expressions of iNOS and COX-2 and β-actin proteins were determined by Western blot **(a)**. The productions of NO and PGE_2 _in the culture supernatants were measured by Griess reactions or ELISA as described in Materials and Methods **(b **and **c)**. Chondrocytes treated with various concentrations of resveratrol for 48 h were collected and MTT assays were performed to determine possible drug cytotoxicity **(d)**. The values shown in each Western blot represented fold inductions of the densitometric intensity compared to the un-stimulated sample after normalization to internal β-actin density. The representative data out of three independent experiments are shown. *: *P *< 0.05.

Because both iNOS-NO and COX-2-PGE_2 _pathways are critical in AGEs-induced cartilage damage [17], we determined whether resveratrol could provide any protective effect through regulating these pathways. The AGEs preparation using methylglyoxal-modified albumin that contains CEL and other AGEs was chosen as the stimulant [18]. When chondrocytes were pretreated with various concentrations of resveratrol, the AGEs-induced expression of iNOS was suppressed (Figure 1a). Meanwhile, resveratrol also inhibited AGEs-induced COX-2 expression. The concentrations of NO and PGE_2_, both downstream products of iNOS and COX-2, respectively, were measured. The results show that resveratrol inhibited AGEs-induced NO and PGE_2 _production (Figure 1b, c). Importantly, resveratrol at a low concentration 25 μM was enough to inhibit PGE_2 _production (Figure 1c). By both MTT and LDH release assays, we confirmed that at the examined concentrations, resveratrol did not have detectable cytotoxic effects in chondrocytes (Figure 1d and data not shown).

### Resveratrol suppressed AGEs-stimulated IKK-IκBα-NF-κB signaling

**Figure 2:**
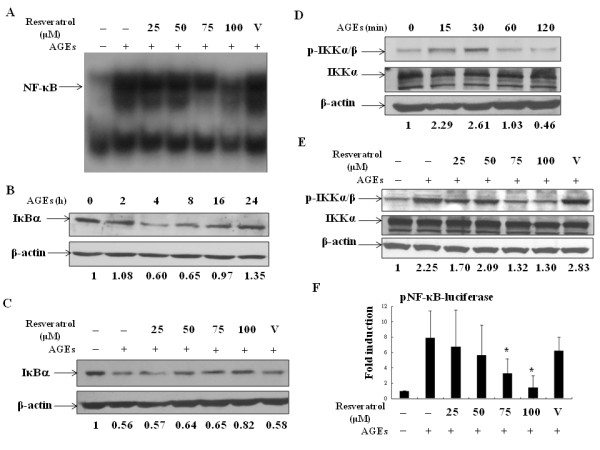
**Resveratrol suppressed AGEs-stimulated NF-κB signaling**. Chondrocytes were cultured overnight with serum-free medium and then pretreated with various doses of resveratrol or the solvent (V) for 24 h and then stimulated with 100 μg/ml AGEs for another 24 h. The cells were collected and the nuclear extracts were prepared for determination of the DNA-binding activity of NF-κB by EMSA **(a)**. In **(b)**, the total cell lysates from chondrocytes treated with AGEs for various time points were collected for determination of the levels of IκBα by Western blot. Similar to (B), except that the cells were pretreated or not with resveratrol before the stimulation with 100 μM AGEs for 4 h **(c)**. In **(d)**, similar to (B), the levels of phosphorylated IKKα/β and total IKKα were determined by Western blot using specific antibodies that recognize phosphorylated IKKα/β or unphosphorylated IKKα. In **(e)**, similar to (D), the effects of resveratrol on the levels of phosphorylated IKKα/β and total IKKα induced by AGEs treatment for 30 min were determined. In **(f)**, the cells were co-transfected with 1 μg of the pNF-κB-luciferase reporter plasmid and the internal control plasmid pTK-Renilla-luciferase at a ratio of 100:1. After transfection, the cells were equally distributed for individual conditions and then pretreated or not with different concentrations of resveratrol for 24 h and then treated with 100 μg/ml AGEs for another 24 h. The cells were collected and the total cell lysates were prepared for luciferase activity determinations. The results were shown as fold inductions of luciferase activity as compared to the unstimulated sample. Values are means ± standard deviations (error bars) for three independent experiments. *: *P *< 0.05 compared to the AGEs-stimulated in the absence of resveratrol treatment.

We next investigated the mechanisms of resveratrol-mediated inhibition of iNOS and COX-2 in AGEs-activated chondrocytes. We focused on examining the activity of NF-κB and AP-1, two families of transcription factors that regulate the activation of iNOS and COX-2 genes and are critical in many inflammatory responses [28,29]. Pretreatment with resveratrol resulted in a dose-dependent inhibition of AGEs-induced DNA-binding activity of NF-κB (Figure 2a). Because the process of IκBα degradation proceeds before NF-κB activation, we examined whether resveratrol had any effect on IκBα degradation. The results show that AGEs treatment caused degradation of IκBα with the effect observed 2 h after treatment (Figure 2b). In the presence of resveratrol, there was dose-dependent reversal of the IκBα levels to a certain extent (Figure 2c). Since degradation of IκBα requires its phosphorylation by IκB kinases, we evaluated whether resveratrol could inhibit IKKα/β activities. By Western blotting with antibodies specifically recognizing phosphorylated forms of both IKKα and IKKβ, the results reveal that AGEs effectively induced phosphorylation of IKKα/β but had no effect on total IKKα (Figure 2d). In the presence of resveratrol, the levels of AGEs-induced phosphorylated forms of IKKα/β decreased (Figure 2e). To determine whether suppression of IKKα/β-IκBα-NF-κB DNA-binding activity could finally lead to decreased transcriptional activation in target genes, luciferase reporter assays were applied. The reporter plasmid pNF-κB-luciferase and the internal control plasmid pTK-Renilla-luciferase were co-transfected into chondrocytes. After transfection, chondrocytes were equally distributed and were stimulated with AGEs in the presence or absence of various doses of resveratrol. Total cell lysates were collected for luciferase activity measurement. We show that consistent with suppression of DNA-binding activity, resveratrol effectively reduced AGEs-induced NF-κB transcriptional activity in a dose-dependent manner (Figure 2f).

### Down-regulation of AGEs-activated AP-1 signaling by resveratrol

**Figure 3:**
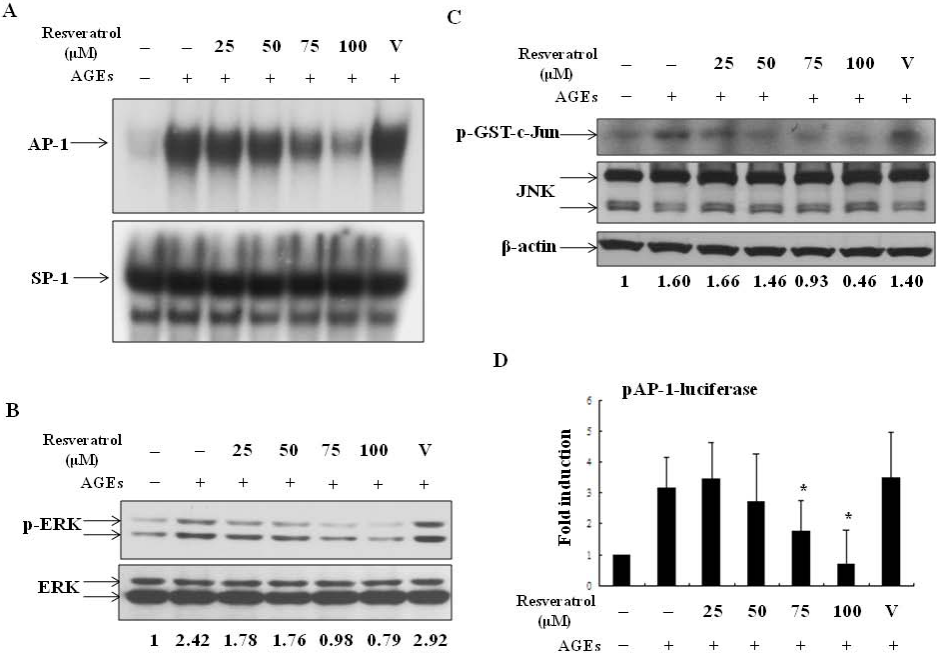
**Resveratrol suppressed AGEs-stimulated AP-1 signaling**. Similar to Figure 2A, Chondrocytes pretreated with various doses of resveratrol were stimulated with 100 μg/ml AGEs and the cells were collected and the nuclear extracts were prepared for determining DNA-binding activity of AP-1 and SP-1 by EMSA **(a)**. The total cell lysates from chondrocytes treated with 100 μg/ml AGEs for 4 h in the presence or absence of pretreatment with various doses of resveratrol were prepared to measure the levels of both un-phosphorylated and phosphorylated ERKs by Western blot **(b)**. In **(c)**, cells were treated in the presence or absence of resveratrol and the total cell lysates were prepared and then immune-precipitated with anti-JNK antibodies. The kinase assays were performed with GST-c-Jun as the substrate. The protein levels of total JNK and β-actin determined by Western blot were presented as controls. In **(d)**, the effects of resveratrol on AGEs-induced AP-1 transcriptional activity were determined. The results were shown as fold inductions of luciferase activity as compared to the unstimulated sample. Values are means ± standard deviations (error bars) for three independent experiments. *: *P *< 0.05 compared to the AGE-stimulated in the absence of resveratrol treatment.

In parallel to its suppressive effects on NF-κB activation, we demonstrated that resveratrol inhibited AGEs-induced AP-1 DNA-binding activity (Figure 3a). As a comparison, resveratrol did not affect SP-1 DNA-binding activity. Since AGEs can induce activation of both ERK and JNK but not p38 in porcine chondrocytes [17], we determined whether resveratrol had any effect on these AP-1 upstream kinase activities. The results show that AGEs-induced phosphorylation of ERK was noticeably suppressed by resveratrol (Figure 3b). In addition, by immunoprecipitation kinase assays, we demonstrated that resveratrol could suppress JNK activity in a dose-dependent manner (Figure 3c); however, resveratrol did not affect the total amount of JNK. Instead, treatment with resveratrol effectively reduced AGEs-stimulated transcriptional activity of AP-1 (Figure 3d).

### Resveratrol affected AGEs-regulated MMP-13 expression and activity and collagen II level

**Figure 4:**
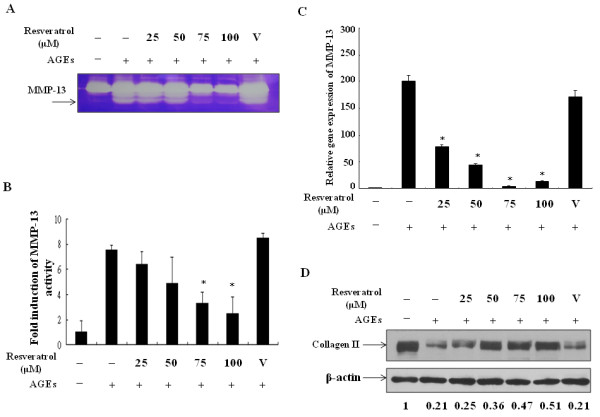
**Resveratrol suppressed AGEs-induced MMP-13 activation and prevented AGEs-mediated collagen II destruction**. Chondrocytes were pretreated with various doses of resveratrol for 24 h and then stimulated with 100 μg/ml AGEs for another 24 h. The activities of MMP-13 released into the culture supernatants were determined by gelatin zymography as described in Materials and Methods **(a)**. The results from three independent experiments were shown **(b)**. In **(c)**, after treatment, the total cellular RNA was prepared and real time RT/PCR was applied to measure the levels of MMP-13 mRNA. In **(d)**, chondrocytes were pretreated with various doses of resveratrol for 24 h, stimulated with 100 μg/ml AGEs for another 24 h and then the levels of collagen II in total cell lysates were determined by Western blot. *: *P *< 0.05 compared to the AGEs-stimulated in the absence of resveratrol treatment.

Different from rheumatoid arthritis, in which infiltrating inflammatory cells and synovial pannus are responsible for joint damage, chondrocytes by themselves are primarily the cells causing cartilage damage through generating and secreting cartilage-breakdown enzymes like MMPs. Because MMP-13, regulated by NF-κB and AP-1 signaling pathways [30], is directly responsible for damaging cartilage matrix, we examined the effects of resveratrol on AGEs-induced MMP-13 activity. As shown in Figure 4a, b, AGEs-induced MMP-13 enzyme activities were suppressed by resveratrol. By quantitative real time RT/PCR analysis, we demonstrate that resveratrol significantly decreased the levels of MMP-13 induced by AGEs treatment (Figure 4c). Since cartilage collagen II is preferentially cleaved by MMP-13, we determined whether resveratrol could affect AGEs-mediated destruction of collagen II. As shown in Figure 4d, resveratrol treatment prevented AGEs-mediated decreases of collagen II by Western blotting. Furthermore, as shown in Additional file 1, resveratrol also suppressed the expression of iNOS and COX-2, the production of PGE_2 _and NO, the DNA-binding activities of NF-κB and AP-1 as well as the enzyme activity of MMP-13 in chondrocytes stimulated by another preparation of AGEs using glycoaldehyde-modified albumin that contains CML, pentosidine and other AGEs [19,20]. Altogether, the results indicate that resveratrol was able to provide cartilage protection at least by down-regulating AGEs-induced MMP expression and enzyme activity as well as by preventing AGEs-mediated decrease of collagen II in chondrocytes.

### Resveratrol protected against AGEs-induced proteoglycan and aggrecan degradation in cartilage explants

**Figure 5:**
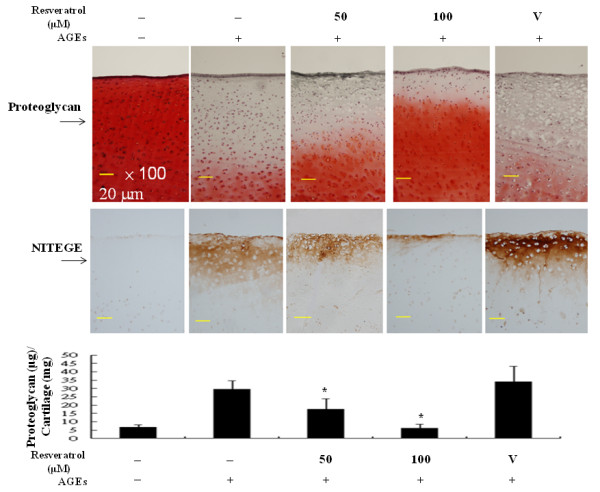
**Resveratrol protected against AGEs-induced degradation of proteoglycan and aggrecan**. Porcine cartilage blocks were cultured in 24-well plates and pretreated or not with 50 or 100 μM resveratrol for 24 h and then stimulated or not with 100 μg/ml AGEs for another 72 h. The retained proteoglycan in cartilage explants was monitored by Safranin O staining (upper panel). In parallel, the intensity of aggrecan staining was examined (middle panel). Meanwhile, the release of proteoglycan into the culture medium was determined and normalized to the cartilage weight as described in Materials and Methods (lower panel). The representative data from three independent experiments using different donor cartilage blocks are shown. * denotes the statistical significance (*P *value < 0.05) as compared to the AGEs-stimulated in the absence of resveratrol treatment.

To further address the chondroprotective effect of resveratrol as a reflection of its anti-inflammatory property, we investigated whether resveratrol could regulate AGEs-induced degradation of cartilage matrix. When the cartilage explants were stained, a significant reduction of Safranin O-positive proteoglycan and an increase of cleavage products of aggrecan (NITEGE) became evident in AGEs-treated samples. Such findings were successfully prevented by resveratrol treatment (Figure 5, upper and middle panels). Consistently, we demonstrated that treatment with resveratrol was effective to prevent AGEs-mediated release of proteoglycan into the supernatants of culture of cartilage explants (Figure 5, lower panel).

## Discussion

Aside from pain-control and rehabilitation, there has been no effective therapeutic regimen for joint disorders in OA patients. One of the major reasons is because of OA's characteristics of long-lasting, slow-progression and the varied-intensity of inflammation. Agents with strong immunosuppressive effects are not good choices in considering the potential adverse events from taking such medications. Thus, the drug of choice for OA should be the one that preserves modest anti-inflammatory properties yet has limited or very low toxicity. The components from food or drink consumed in our regular daily activity may be reasonable choices. We therefore, in this study, investigated the potential anti-inflammatory action of resveratrol in AGEs-stimulated chondrocytes.

AGEs derived from an array of precursor molecules are very heterogenous in chemical structures. Three major well-characterized AGEs, including pentosidine, CML, and CEL all increase with age in cartilage collagen [31]. Both the sum of these AGEs (CML, CEL and pentosidine) and the AGEs fluorescence correlate significantly with the mean degree of modification of arginine, hydroxylysine and lysine residues in collagen [31]. The concentrations of AGEs products are five-fold higher in elderly OA patients than those in younger patients [32,33]. In persons aged 20 to 80, there is a good linear correlation of increases of fluorescent intensities of accumulated AGEs in cartilage and the difference sometimes can be more than 11-fold higher [31,34]. The fluorescence intensities in AGEs at concentrations ranging from 25 to 100 μg/ml were two- to six-fold higher compared to those in BSA and therefore were close to the conditions in aged joints of human beings (data not shown).

**Figure 6:**
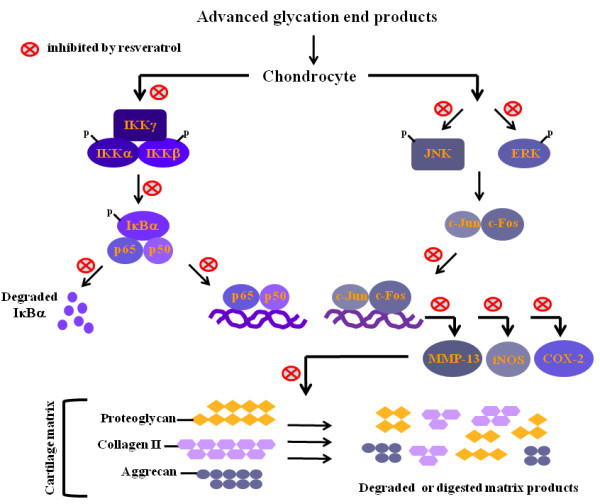
**The effects and mechanisms of resveratrol in AGEs-mediated damage of cartilage**. AGEs stimulation activated both NF-κB and AP-1 signaling pathways. The activation of NF-κB and AP-1 signaling leads to the generation of iNOS, COX-2 and MMP-13 that cause the degradation of proteoglycan, aggrecan and collagen II, three major components of cartilage matrix, through direct and indirect mechanisms. Along the activation of these critical pathways by AGEs, many associated events were blocked by the treatment of resveratrol as indicated.

When the physiological concentration of AGEs was examined in this study, the results show that resveratrol could suppress AGEs-induced iNOS-NO and COX-2-PGE_2 _production and the mechanisms were likely through inhibiting IKK-IκBα-NF-κB and JNK/ERK-AP-1 signaling pathways. Such anti-inflammatory properties of resveratrol could be faithfully reflected by detecting the degradation of proteoglycan and aggrecan as well as the suppression of proteoglycan release from cartilage explants, the inhibition of collagen II decrease and the attenuation of MMP expression and enzyme activity induced by AGEs (summarized in Figure 6). This study thus provides evidence of chondroprotective effects and mechanisms of resveratrol as well as its potential application in AGEs-mediated pathogenesis in OA joints.

Among the factors contributing to OA pathogenesis, both COX-2 and iNOS play important roles [35,36]. Several reports indicate that the inhibition of NO production can suppress IL-1-induced chondrocyte apoptosis and reduce the severity of cartilage damage in experimental OA models [37,38]. Given the evidence that activation of the COX-2-PGE_2 _pathway in chondrocytes results in cell apoptosis, Pelletier *et al. *[39] showed that the inhibition of COX-2 rescues cells from death in experimental OA of dogs. Treatment with non-steroidal anti-inflammatory drugs also inhibits IL-1-induced MMP-1 and MMP-3 production in bovine articular chondrocytes [40]. The importance of both iNOS and COX-2 in AGEs-mediated pathologies is further supported by our report that successful blockage of either COX-2 or iNOS activity significantly reduces AGEs-mediated proteoglycan release and cartilage damage [17]. The data from Dave *et al. *[13] also reveal that resveratrol suppresses IL-1β-induced COX-2-PGE_2 _activation and mediates anti-apoptosis effects in human articular chondrocytes. Accordingly, the inhibition of both COX-2-PGE_2 _and iNOS-NO pathways by resveratrol should suggest that it is a potential chondroprotective regimen.

Consistent with the effects on COX-2 and iNOS, the AGEs-induced DNA-binding and transcriptional activity of NF-κB and AP-1, which also regulate the enhancer/promoter regions of these two enzymes, were susceptible to the suppression by resveratrol. In addition, the NF-κB and AP-1 upstream signaling molecules, including IKK-IκBα and ERK/JNK, were suppressed by resveratrol. Somewhat different from its effect on IL-1-induced upstream molecules regulating NF-κB activation, reseveratrol suppressed AGEs-induced but not IL-1-induced IKK activity [12]. These observations further distinguish the down-stream signaling events regulated by AGEs stimulation from those induced by the pro-inflammatory cytokine IL-1. Given broad spectrums of NF-κB- and AP-1-targeted downstream events, resveratrol may exert many more therapeutic benefits in AGEs-mediated pathogenesis other than the evidence demonstrated in this report. In support of this conclusion, resveratrol has been shown to down-regulate the expression of pro-inflammatory mediator and cytokine genes via inhibiting NF-κB signaling pathway [14]. The results from this study also highlight the potential of examining small molecule like JNK or ERK inhibitors as suggested by other researchers in ageing-related disorders caused by deposition and accumulation of AGEs [41].

In this study, we further show that aside from the suppression of iNOS-NO, COX-2-PGE_2_, IKK-IκBα-NF-κB and JNK/ERK-AP-1 signaling pathways, resveratrol could potently inhibit the expression and enzyme activity of MMP-13. The results were consistent with earlier studies demonstrating that the activation of MMP-13 critically requires the transcriptional activity of both NF-κB and AP-1 [30]. According to Mitchell *et al. *[42], MMP-13 expression co-localizes with collagen II degradation in active OA lesions and the inhibition of MMP-13 greatly reduces collagen II degradation. In contrast, the increased expression of MMP-13 in cartilages and joints using tetracycline-regulated transcription in conjunction with a cartilage-specific promoter results in exaggerated loss of proteoglycan and cleavage of collagen II in a transgenic animal model [43]. In addition to being the most potent collagen II-degrading enzyme, MMP-13 can degrade proteoglycan and aggrecan and play a dual role in damaging cartilage matrix [44,45]. Altogether, the results presented in this report suggest that the treatment with resveratrol helps to protect against AGEs-induced degradation of cartilage components like proteoglycan, aggrecan and collagen II.

There are two major limitations in this study. First, the results of examining porcine chondrocytes could not be exactly translated into those in studying human chondrocytes although the study of choncrocytes from different species has been frequently applied by many researchers. Secondly, the concentrations of resveratrol examined in this study were relatively high. A "good" red wine contains approximately 75 μmol/L content of resveratrol, which makes reasonable wine consumption unlikely to reach effective anti-inflammatory concentrations in plasma in human bodies [46]. Because of its lipophilic character, resveratrol has been shown to accumulate in tissues such as the heart, liver, and kidney [47]. It is therefore concluded that long term absorption of a sufficient quantity of resveratrol may increase the beneficial effect of red wine on health [48]. Indeed, *in vitro *approaches demonstrate that high concentrations of resveratrol (10 to 100 μM) have cardioprotective effects by inducing endothelial nitric-oxide synthase gene expression and increasing nitric oxide levels in cultured endothelial cells [46]. High concentrations of resveratrol also inhibit interleukin-5-induced activation of human peripheral blood eosinophils without reduction of cell viability [49]. One of the therapeutic approaches with high concentrations of resveratrol (10 to 100 μM) in the treatment of AGEs-induced cartilage damage may be performed through local delivery that gains high anti-inflammatory and intra-articular concentrations and provides protection of cartilage from damage by AGEs. Further studies are being performed to investigate this possibility in OA animal models.

## Conclusions

In this study, we show that aside from the suppression of iNOS-NO, COX-2-PGE_2_, IKK-NF-κB and MAPK-AP-1 signaling pathways, resveratrol could potently inhibit MMP-13 expression. All these observed effects by resveratrol contributed to the protection against AGE-induced degradation of cartilage components like proteoglycan, aggrecan and collagen II (Figure 6). Understanding the molecular mechanisms activated by accumulated AGEs in joints and how the processes may be modified by resveratrol could provide additional approaches currently unavailable to slow the progression of OA. It is also anticipated that the results from this report will bring more *in vitro *and *in vivo *studies to confirm the therapeutic benefits of resveratrol in patients with OA and AGEs-mediated disorders.

## Abbreviations

AGEs: advanced glycation end products; AP-1: activator protein-1; BSA: bovine serum albumin; COX-2: cyclooxygenase-2; DMB: 1,9-dimethylmethylene blue; DMEM: Dulbecco's modified Eagle's medium; ELISA: enzyme-linked immunosorbent assay; EMSA: electrophoretic mobility shift assays; ERK: extracellular signal-regulated kinase; FBS: fetal bovine serum; GAG: glycosaminoglycan; IKK: IκB kinase; IL-1: interleukin-1; iNOS: inducible nitric oxide synthase; JNK: c-Jun N-terminal kinase; MMP: matrix metalloproteinase; MTT: Dimethylthiazol-2-y)-2,5-diphenyl-tetrazolium bromide; NF-κB: nuclear factor-kappaB; OA: osteoarthritis; PBS: phosphate-buffered saline; PGE_2_: prostaglandin E_2_; PMSF: phenylmethylsulphonyl fluoride; SDS-PAGE: sodium dodecyl sulphate-polyacrylamide gel electrophoresis; TNF-α: tumor necrosis factor alpha.

## Competing interests

The authors declare that they have no competing interests.

## Authors' contributions

FCL designed the study, performed most of the experiments, wrote the manuscript and took responsibility for the correctness of the study results. JHL and LJH critically corrected the manuscript, gave advice and guided experimental steps along the experimental process. WLW and DMC helped to perform part of the cartilage explants cultures, histological analysis, proteoglycan measurements and reviewed the manuscript. All authors read and approved the final manuscript.

## Supplementary Material

Additional file 1 **Figure S1**. Effects of resveratrol on chondrocytes activated by an AGEs preparation using glycoaldehyde-modified albumin (gAGEs) that contains N(epsilon)-carboxymethyllysine (CML), pentosidine and other AGEs. Click here for file
