# Supplementary material for: Chondroprotective effects and mechanisms of resveratrol in advanced glycation end products-stimulated chondrocytes
Source: Arthritis Res Ther. 2010 Sep 8;12(5):R167. doi: 10.1186/ar3127 (PMC2990994; doi:10.1186/ar3127)
Supplement: Additional file 1 — Figure S1. Effects of resveratrol on chondrocytes activated by an AGEs preparation using glycoaldehyde-modified albumin (gAGEs) that contains N(epsilon)-carboxymethyllysine (CML), pentosidine and other AGEs. [file ar3127-S1.DOC]

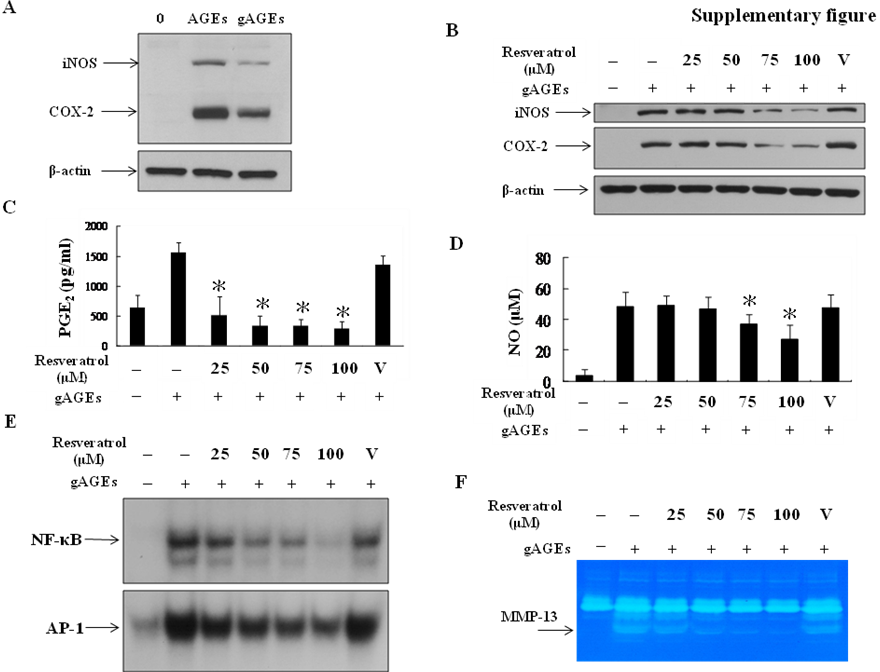


Effects of resveratrol on chondrocytes activated by an AGEs preparation using glycoaldehyde-modified albumin (gAGEs) that contains N(epsilon)-carboxymethyllysine (CML), pentosidine and other AGEs. (A) showed the induction of iNOS and COX-2 by two different preparations of AGEs. Labeled as AGEs is the major AGEs used for most of this study. The results also indicated that resveratrol inhibited gAGEs-induced expression of iNOS and COX-2 (B), production of PGE_2_ (C) and NO (D), DNA-binding activities of NF-κB and AP-1 (E) as well as enzyme activity of MMP-13 (F). Chondrocytes were pretreated with various doses of resveratrol or the solvent (V), ethanol, and then stimulated with gAGEs (100 μg/ml) exactly as the conditions described for the other preparation of AGEs in this study. The expressions of iNOS, COX-2 and β-actin were determined by Western blot (A and B). The productions of PGE_2_ and NO in the culture supernatants were measured by ELISA and Griess reactions, respectively (C and D). The DNA-binding activities of NF-κB and AP-1 were determined by EMSA (E). The enzyme activity of MMP-13 was measured by gelatin zymography (F). The representative data out of at least three independent experiments are shown. *: P < 0.05.
